# Non-Invasive Characterization of Different *Saccharomyces* Suspensions with Ultrasound

**DOI:** 10.3390/s24196271

**Published:** 2024-09-27

**Authors:** Dominik Geier, Markus Mailänder, Iain Whitehead, Thomas Becker

**Affiliations:** Chair of Brewing and Beverage Technology, TUM School of Life Sciences, Technical University of Munich, 85354 Freising, Germanyiain.whitehead@tum.de (I.W.); tb@tum.de (T.B.)

**Keywords:** ultrasonic characterization, non-invasive measurement, yeast suspensions, *Saccharomyces*, yeast concentration, cell count

## Abstract

In fermentation processes, changes in yeast cell count and substrate concentration are indicators of yeast performance. Therefore, monitoring the composition of the biological suspension, particularly the dispersed solid phase (i.e., yeast cells) and the continuous liquid phase (i.e., medium), is a prerequisite to ensure favorable process conditions. However, the available monitoring methods are often invasive or restricted by detection limits, sampling requirements, or susceptibility to masking effects from interfering signals. In contrast, ultrasound measurements are non-invasive and provide real-time data. In this study, the suitability to characterize the dispersed and the liquid phase of yeast suspensions with ultrasound was investigated. The ultrasound signals collected from three commercially available *Saccharomyces* yeast were evaluated and compared. For all three yeasts, the attenuation coefficient and speed of sound increased linearly with increasing yeast concentrations (0.0–1.0 wt%) and cell counts (R^2^ > 0.95). Further characterization of the dispersed phase revealed that cell diameter and volume density influence the attenuation of the ultrasound signal, whereas changes in the speed of sound were partially attributed to compositional variations in the liquid phase. This demonstrates the ability of ultrasound to monitor industrial fermentations and the feasibility of developing targeted control strategies.

## 1. Introduction

The remarkable biodiversity of yeast leads to extensive industrial applications, including brewing, baking, and industrial biotechnology [1,2,3]. *Saccharomyces cerevisiae*, commonly known as brewer’s or baker’s yeast, is used in the production of many traditional fermented foods and beverages, such as bread, beer, and wine. In brewing, yeast is essential for converting fermentable sugars into alcohol and carbon dioxide. In addition, the flavor and aroma profiles of the finished beers depend on the *Saccharomyces* species and strain used, as well as the process parameters (e.g., temperature regime and aeration) during production. By contrast, in baking, yeast is used for leavening dough, resulting in the characteristic airy texture of bread. Moreover, in biotechnology, the versatile genetic profile and multiple biochemical pathways make yeast a flexible microorganism for the production of bioactive substances and microbial proteins [4]. Species-specific properties determine the required fermentation conditions as well as the quality of the finished products. Consequently, microorganisms are selected based on their unique characteristics, such as budding and flocculation behavior, and other physiological properties, e.g., substrate requirements. Yeast morphology directly influences the performance and can vary significantly, particularly in terms of cell size, shape, budding behavior, and surface properties [5,6,7]. In fermented beverage production (e.g., beer and wine), yeast biomass concentration has a direct impact on the rate of substrate conversion, product formation, and overall process efficiency [8]. Therefore, monitoring both the composition of the dispersed solid phase (biomass or cell count) and the continuous liquid phase of a biological suspension (nutrients and metabolites) is crucial for maintaining favorable conditions throughout the process.

The different methods commonly used to measure yeast cell concentration in solutions offer several individual advantages but also suffer from specific limitations. Turbidity measurements estimate cell density based on light absorbance; these are widely used in offline and in-line applications due to their simplicity and cost-effectiveness. However, their accuracy can be compromised by the yeast properties, including cell size, shape, aggregation, and concentration [9]. In highly concentrated or colored suspensions, the low linear range of turbidity sensors needs sample dilution to maintain accuracy [10,11]. Furthermore, probe fouling reduces sensor performance over time [12]. Conversely, gravimetric biomass concentration determination is a laborious and time-consuming offline technique requiring sufficient biomass concentration to yield reliable results. When working at low concentrations, large sample volumes are needed [13]. Another commonly used method to determine the cell count and cell properties is flow cytometry. However, this method is time-consuming and labor-intensive, requires skilled personnel, and incurs considerable costs. Flow cytometers detect the scatter of a light beam focused on a stream of aligned single cells [13,14]. In contrast, Coulter counters measure cell concentration based on changes in electrical resistance as cells pass through a small orifice [15]. However, the change in electrical resistance is susceptible to the chemical composition of the medium, which can vary due to cell metabolism, leading to potential inaccuracies [16]. Moreover, to ensure that only single cells enter the measuring module, the method requires sample dilution, making it unsuitable for continuous, real-time monitoring [17]. Lastly, despite being manual and time-consuming, microscopy-based counting methods remain the standard for verifying results obtained from automated systems [18]. In these manual methods, cell count is determined using a microscope and hemocytometer. Although minimal instrumental equipment is required, this method is offline and ex situ and demands significant practical expertise to yield reliable data [13]. Common limitations to all methods include being invasive and time-consuming and providing limited insight into the dynamic properties of yeast suspensions. In addition, methods requiring sampling introduce a contamination risk and a time lag relative to the process, thus often failing to reflect the actual conditions of a bioprocess [19]. Consequently, there is a growing interest in developing non-invasive, real-time techniques that can overcome these challenges.

Raman spectroscopy and in situ microscopy (ISM) are in-line techniques used for monitoring bioprocesses and measuring cell concentration in biological suspensions. In ethanol fermentations, Raman spectroscopy significantly enhanced ethanol yield by maintaining optimal process conditions [20,21]. Although Raman spectroscopy is valuable for in-line bioprocess monitoring, it faces considerable challenges, particularly since the Raman signal is inherently weak and easily masked by noise, making accurate spectral analysis difficult [22,23]. Fluorescence—originating from various biomolecules—is often much stronger and superimposes the Raman signal, particularly when measurements are taken at high concentrations [24,25]. Furthermore, Raman spectroscopy is costly due to the requirement for high-precision equipment, complex data analysis, and integration into existing systems, all of which contribute to its substantial capital expenditure and operating expense [26]. In situ microscopy also allows real-time monitoring of yeast cell concentrations in bioprocesses and provides continuous data that can be used to optimize growth conditions and improve process efficiency [27]. However, the sensitivity of ISM decreases at higher cell concentrations, and its effectiveness is constrained to concentrations below 4.5 × 10⁶ cells/mL [28]. Additionally, the technique is limited by optical resolution when dealing with small or poorly contrasted cells [29].

Ultrasound is a real-time, in-line method recognized for its non-invasive and non-destructive nature; typically, ultrasound signals are externally generated and transmitted into the sample for measurement. Unlike Raman spectroscopy and ISM, ultrasound can examine opaque media and high concentrations. The generated ultrasonic spectra interact with insoluble and soluble substances in a suspension [30,31]. The interaction between the sample and the ultrasound signal is frequently determined by attenuation, which represents the reduction in the amplitude of the signal. Additionally, variations in the propagation speed of the signal (i.e., speed of sound or ultrasonic velocity) are also commonly recorded [32,33]. Therefore, ultrasonic techniques are extensively investigated to evaluate and characterize complex aqueous solutions and provide information on the composition of the dispersed solid phase and the liquid phase.

Several studies have focused on the development of ultrasonic methods for the characterization of the insoluble, dispersed solid phase of aqueous systems. Goodenough et al. [34] used ultrasound spectroscopy to investigate the attenuation of aqueous solutions containing particulate samples (e.g., industrial particles and spherical silica). They found that the attenuation coefficient is directly proportional to the concentration of insoluble particles. Later, Chen et al. [35] developed a 50 MHz ultrasound system for measuring ultrasonic backscattering and correlated it with the concentration of red blood cells. Similarly, Elvira et al. [36] employed high-frequency ultrasound backscattering to determine yeast suspension concentrations. In their laboratory setup, 5 µL samples were placed in a Neubauer grid and analyzed with ultrasonic pulse-echo measurements. Rodriguez-Molares et al. [37] proposed an empirical model based on ultrasonic attenuation, temperature, and frequency to estimate the biomass concentration of cyanobacteria. However, their method was invasive, utilizing a pair of ultrasonic transducers immersed in the suspension, rendering it unsuitable for bioprocess control due to hygienic risks. Zhan et al. [38] employed an ultrasonic measurement system combined with least squares support vector machines for online measurement of titanium dioxide particles in multi-component suspensions. Recently, Akbari et al. [39] developed a non-invasive acoustic sensor using ultrasonic pulsed Doppler to monitor the concentration of Chinese hamster ovary cells in real-time. These studies further confirm ultrasonic techniques can be used to analyze and estimate the dispersed solid phase of a suspension.

In sharp contrast, the analysis of the liquid phase focuses on exploiting changes in the speed of sound to determine the concentrations of the soluble substances [40,41]. Schöck et al. [40] focused on developing an ultrasonic sensor array to detect sucrose and ethanol in a ternary mixture of water, ethanol, and sucrose. They determined that ultrasonic velocity correlates linearly with the dissolved sugar concentrations in aqueous solutions. The influences of sucrose and ethanol on ultrasonic velocity were additive but non-linear across different temperatures and solvent concentrations. However, ethanol and sucrose concentrations needed to be determined by measuring ultrasonic velocity at two distinct temperatures [40]. Density and speed of sound measurements were also conducted as a function of mass concentration in binary solutions of glucose-water, fructose-water, sucrose-water, and ethanol-water [42]. Vatandas et al. [43] demonstrated that ultrasonic velocity transmission measurements can non-destructively evaluate ethanol at specific volume fractions. Hoche et al. [44] systematically studied the ternary system water–maltose–ethanol, focusing on critical process parameters such as density, speed of sound, and temperature. They demonstrated the feasibility of predicting online concentrations in multi-component mixtures of polar liquids by determining density and ultrasonic velocity. Celik et al. [45] monitored lactic acid using ultrasonic velocity measurements, while Krause et al. [46] employed several acoustic features calculated from time and frequency domain representations, combined with multivariate calibration, to determine the sugar concentration in maltose-water mixtures.

To date, the application of ultrasonic techniques for measuring the concentrations of substances in aqueous mixtures containing both insoluble and soluble compounds has been scarcely investigated. Geier et al. [47] investigated attenuation and speed of sound measurements of yeast-maltose suspensions and discussed its potential for bioprocess monitoring. Zahn et al. [48] examined titanium dioxide-glucose mixtures using a comparable approach. In both studies, the attenuation coefficient was directly proportional to the concentration of insoluble particles but remained independent of the concentration of soluble substances, likely due to the relatively low concentration of soluble substances. Furthermore, the ultrasound measurements may vary significantly depending on the composition and concentration of the insoluble particles, particularly those with heterogeneous dimensions and acoustic properties, which were not investigated. Therefore, the effects of cell size diversity and varying acoustic properties in biological suspensions remain largely unexplored. Further research is required to investigate how these factors influence ultrasound signals and instrument performance, leading to improved monitoring strategies for bioprocesses.

Given the extensive industrial use of yeast strains with varying properties, in this study, three commercially available yeasts with distinct physiological properties were selected. Two top-fermenting yeasts, *Saccharomyces cerevisiae* and WB-06 (*Saccharomyces cerevisiae var. diastaticus*), were examined; the former is typically used in bread production, and the latter is for wheat beers (e.g., Hefeweizen and Belgian Witbier). A bottom-fermenting yeast, W 34/70 (*Saccharomyces pastorianus*), extensively used for lager beer production [49], was also investigated. This study evaluates the response of different *Saccharomyces* strains to ultrasonic measurements. First, ultrasound was used to analyze yeast suspensions with increasing yeast concentration (weight percent; wt%); these covered the range typically used in industry applications [50]. The cell count (cells/mL), as well as the attenuation coefficients and speed of sound, were determined for all samples in the concentration gradient (0.0–1.0 wt%). Subsequently, to explore the effect of yeast cells on the ultrasound signals, all 1 wt% yeast suspensions and their corresponding filtrates were investigated and compared. In addition, the impact of particle size distribution on the attenuation coefficients was assessed as well as the influence of liquid phase composition on speed of sound. This analytical approach allowed to simultaneously assess the influence of the dispersed solid phase and continuous liquid phase on ultrasound signals in a biological suspension.

## 2. Materials and Methods

### 2.1. Preparation of Yeast Suspensions and Filtrates

In Table 1, the three commercially available dry yeast strains used in this study are listed. All closed dry yeast samples were stored at +4 °C until use.

**Figure 1:**
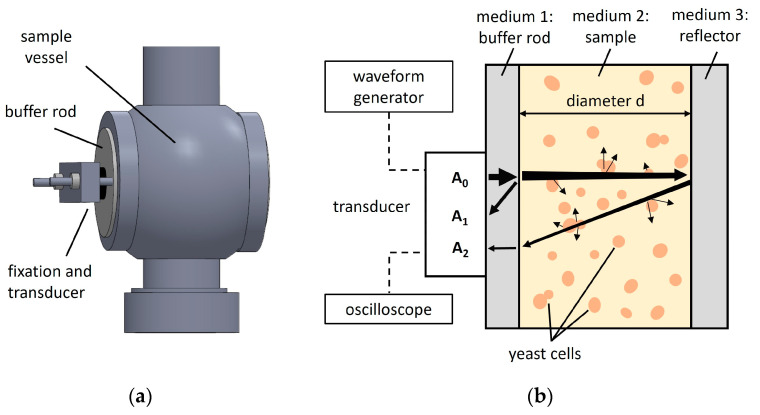
(**a**) Technical drawing of the pulse-echo measurement setup, including the sample vessel. The buffer rod introduces a time delay in the ultrasound signal. The transducer, securely fixed in position, is responsible for transmitting and receiving the ultrasound signals. (**b**) Schematic representation of the ultrasound signal propagation in the pulse-echo measurement setup. Arrows represent the ultrasound signal and its interaction with the media. The waveform generator excites the transducer, generating the initial ultrasound wave, A_0_. The ultrasound signal then travels through the buffer rod into the sample and is reflected at the buffer rod–sample interface (first reflection) and the sample–reflector interface (second reflection). These reflections are received by the transducer as A_1_ and A_2_, respectively, and recorded using the oscilloscope.

For the yeast suspensions, Ringer solution was selected as the dispersion medium because its electrolyte concentration prevents the transport of substances from inside the cell into the solution, thereby maintaining the original physiological state and morphology of the yeast. To prepare a 25% Ringer solution (i.e., electrolyte solution), one Ringer tablet (Merck KGaA, Darmstadt, Germany) was dissolved in 500 mL of demineralized water. The concentration gradient for each yeast suspension ranged from 0.0 to 1.0 wt% and consisted of 11 samples (i.e., 0.1 wt% increments). For each yeast suspension, the weighed dry yeast was suspended in Ringer solution for 60 min, tempered to 20 ± 0.5 °C in a temperature-controlled water bath (Pro-line RP-3530, Lauda Dr. R. Wobser GmbH & Co. KG, Lauda-Königshofen, Germany), and then transferred to the sample vessel (250 mL) of the experimental setup, shown in Figure 1a. In addition, the yeast cell count at each concentration level was determined using a hemocytometer with a depth of 0.1 mm (Blaubrand, Sigma-Aldrich, St. Louis, MI, USA), as outlined in MEBAK 10.4.3.1 and MEBAK 10.11.4.4 [51].

Filtrates of the 1 wt% yeast suspensions were prepared for all yeasts. The dispersed phase (i.e., yeast cells) was removed by filtering the yeast suspensions. Folded filter papers (MN 514, Machery-Nagel GmbH & Co. KG, Düren, Germany) and fine diatomaceous earth (a spatula tip) were used to separate the two phases. For each filtration, the first 50 mL were collected and returned to the filter. This allowed the diatomaceous earth to settle on the folded filter and form a filter layer. The filtrates were then tempered to 20 ± 0.5 °C and transferred to the sample vessel of the experimental setup (Figure 1a). Microscopic sampling confirmed that no yeast cells were present in the filtrates. Additionally, these filtrates were further characterized as described in Section 2.4.

### 2.2. Experimental Ultrasound Setup and Operating Principle

The pulse-echo measurement setup is illustrated in Figure 1a. A Varivent^®^ process connector was utilized as the sample vessel, allowing easy integration into various industrial bioprocesses [47]. The sample vessel consists of stainless steel with two opposing parallel Plexiglas^®^ latches, the buffer rod (i.e., medium 1) and the reflector (i.e., medium 3). The Plexiglas^®^ had a density of 1.17 g/cm^3^ and an ultrasonic velocity of 2760 m/s at 20 °C [52] and a specific attenuation coefficient, *α_B_* [53]. The diameter of the buffer rod, *d_B_*, was 17 mm. The bottom of the Varivent^®^ process connection was sealed with a blind nut. A 10 Vpp pulse was generated using a waveform generator (Agilent 35522A, Keysight Technologies, Santa Rosa, CA, USA) and a four-cycle square excitation. The ultrasound signal was transmitted into the sample (i.e., medium 2) by a 2 MHz ultrasonic transducer with a diameter of 10 mm (MB 2 SE, GE, Boston, MA, USA) affixed to the buffer rod of the sample vessel.

The same transducer received the reflected ultrasound signals, which were recorded using an oscilloscope (PicoScope 5204, Pico Technology, Cambridgeshire, UK) at a 1 GHz sampling rate. Coupling gel (Aquasonic 100, Parker Laboratories Inc., Fairfield, CT, USA) was used to guarantee effective ultrasound transmission.

Figure 1b depicts a cross-sectional scheme of the sample vessel, illustrating the propagation and subsequent reflections of ultrasonic waves within the system. Upon initiation, an ultrasonic wave with an initial amplitude, denoted as A_0_, is generated and transmitted into the buffer rod. As the wave traverses the buffer rod, a portion of its energy is reflected at the interface between the buffer rod and the sample (i.e., yeast suspensions or filtrates), resulting in a first echo with an amplitude of A_1_.

The remaining fraction of the incident wave, A_0_, that successfully enters the sample continues to propagate and interact with the sample until it reaches the reflector. At this point, the wave reflects off the sample-reflector interface, producing a second echo. It is important to note that this second echo undergoes a 180° phase shift, a phenomenon typically observed in reflection processes where there is an impedance mismatch between the contacting materials [54,55]. This second echo is then detected by the transducer with an amplitude denoted as A_2_.

### 2.3. Ultrasound Measurements of Yeast Suspensions and Filtrates

After preparing the concentration gradient and the filtrates for the three yeast suspensions, as described in Section 2.1, these were manually filled in the sample vessel (see Figure 1). To ensure proper cleaning of the sample vessel, hand washing with tap water and double rinsing with distilled water followed by drying was performed. For each sample, 30 ultrasound signals were recorded at 0.5 s intervals, resulting in a total measurement time of 15 s. For each yeast, all samples in the concentration gradient (0.0–1.0 wt%), as well as their filtrates, were measured in technical triplicates (N = 3). The ultrasound signals were processed and analyzed, as explained in Section 2.4.

### 2.4. Ultrasound Signal Processing and Analysis

All recorded ultrasound signals were processed and analyzed using Matlab 2020b (The MathWorks, Inc., Natick, MA, USA).

#### 2.4.1. Determination of Speed of Sound

The speed of sound, c, was calculated by dividing the propagation distance, which is twice the diameter, d, of the sample vessel, by the time of flight (TOF):

(1) $$c=\frac{2d}{TOF}$$

**Figure 2:**
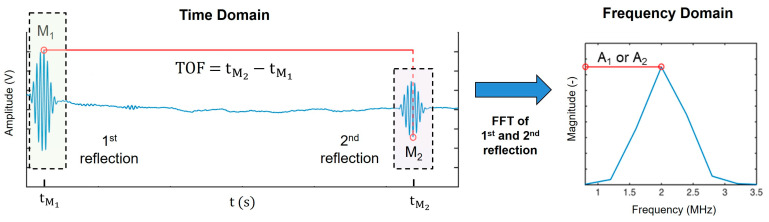
Schematic time and frequency domain analysis of ultrasound signals. The left panel presents the time domain signal, highlighting the maximum amplitude, *M*_1_, of the first reflection and the phase shift resulting in *M*_2_ for the second reflection. The time of flight (*TOF*) is indicated between the first and second reflection and refers to the time of flight of the ultrasound signal through the sample. Following extraction of the first and second reflections, the Fast Fourier Transform (FFT) is applied, as depicted in the right panel. This panel illustrates the frequency domain representation of a first or second reflection, with magnitudes A_1_ or A_2_, respectively.

The TOF was calculated in the time domain of the ultrasound signals as the time difference between the global minimum, $M_{2}$, of the second reflection and the global maximum, $M_{1}$, of the first reflection, as illustrated in Figure 2. The diameter, d, was determined by calibration with a reference liquid (i.e., water) of known speed of sound and temperature [44]:

(2) $$d=\frac{{TOF}_{w,\;20{}^{\circ}C}\;{\;\cdot c}_{w,\;20{}^{\circ}C}}{2}$$

where ${TOF}_{w,20{}^{\circ}C}\;$is the measured time of flight in water at 20 °C and $c_{w,20{}^{\circ}C}$ is the speed of sound of water at 20 °C. The speed of sound of water at 20 °C was calculated with a fifth-order polynomial describing the dependence of the speed of sound in water on temperature, as reported by Marczac [56]:

(3) $$c_{w,\;T}=\sum_{i=0}^{5}a_{i}\cdot T^{i}$$

where T is the temperature in °C and $a_{i}$ are the polynomial coefficients [56]. The calculated diameter, d, of the vessel is 37.5 mm at 20 °C.

#### 2.4.2. Determination of the Attenuation Coefficient

The reflection coefficient, R, measures the fraction of an acoustic wave’s amplitude reflected at an interface of two media with different acoustic impedances. Acoustic impedance represents the resistance an acoustic wave encounters as it propagates through a material [57]. At a boundary between two media with different impedances, $Z_{1}\;$and $Z_{2}$, the reflection coefficient, $R_{12,}$, can be calculated:

(4) $$R_{12}=\frac{Z_{2}-Z_{1}}{Z_{2}+Z_{1}}=\frac{\rho_{2}\cdot \;c_{2}-\rho_{1}\cdot \;c_{1}}{\rho_{2}\cdot \;c_{2}+\rho_{1}\cdot \;c_{1}}$$

where $\rho_{1}$ is the density, $c_{1}$ is the speed of sound of medium 1, $\rho_{2}$ is the density, and $c_{2}$ is the speed of sound of medium 2. The amplitudes, $A_{1}\;$and $A_{2}$, (see Figure 1 and Figure 2), are given by [58]:

(5) $$A_{1}=A_{0}\cdot e^{-{2\alpha }_{B}d_{B}}{\cdot R}_{12}$$

(6) $$A_{2}=A_{0}\cdot e^{-{2\alpha }_{B}d_{B}}\cdot T_{12}\cdot R_{23}\cdot T_{21}\cdot e^{-2\alpha d}$$

$R_{12}$ and $R_{23}$ are the reflection coefficients at the interface buffer rod-sample and sample-reflector, and $A_{0}$ is the incident wave amplitude. The term $e^{-{2\alpha }_{B}d_{B}}$ represents the attenuation by the buffer, where *α_B_* is the attenuation coefficient of the buffer and the propagation distance is twice the buffer diameter, $d_{B}$. The term $e^{-2\alpha d}$ represents the attenuation by the sample, where α is the attenuation coefficient and the propagation distance is twice the diameter, d, of the sample vessel (see Figure 2). Here, $T_{12}$ and $T_{21}$ correspond to the transmission coefficients at the interface buffer rod-sample and at the interface sample-buffer rod, respectively. The incident wave amplitude, $A_{0}$, and the attenuation, $e^{-{2\alpha }_{B}d_{B}}$, were assumed to be constant since the transducer excitation remained constant throughout all experiments and were summarized to a constant $C_{0}$:

(7) $$A_{1}=C_{0}{\cdot R}_{12}$$

(8) $$A_{2}=C_{0}\cdot T_{12}\cdot R_{23}\cdot T_{21}\cdot e^{-2\alpha d}$$

The constant ${C_{0}=A}_{0}\cdot e^{-{2\alpha }_{B}d_{B}}$ is defined by the known buffer diameter, $d_{B}$, the specific attenuation coefficient, $\alpha_{B}$, [53] and $A_{0}$. The value of $A_{0}$ was derived from a reference measurement conducted with water at 20 °C:

(9) $$A_{0}=\frac{A_{1,water}}{e^{-{2\alpha }_{B}d_{B}}\cdot R_{12}}$$

where $R_{12}$ represents the reflection coefficient at the buffer–water interface, calculated using Equation (4), based on the known density and speed of sound of the buffer material and water at 20 °C. The received signal was analyzed, and the amplitude of the first reflection, $A_{1,water}$, was extracted from the discrete Fourier transform as the magnitude at 2 MHz, following the same process used for determining the amplitudes, $A_{1}$ and $A_{2}$, for the yeast suspensions.

To determine the amplitudes, $A_{1}$ and $A_{2}$, the first reflections were aligned at the maximum, *M*_1_, and the second reflections were aligned at the minimum, *M*_2_. Subsequently, the signals were superimposed and extracted from the same position in the time domain record. These extracted signals, consisting of 300 points, were then transformed into the frequency domain via the discrete Fourier transform (see Figure 2). The magnitudes at 2 MHz of the transforms of the first and second reflections, denoted as $A_{1}$ and $A_{2}$, respectively, were derived. The attenuation coefficient can be formulated as [47,57]:

(10) $$\alpha =\frac{1}{2d}\cdot ln\left(\left|\frac{-A_{1}\cdot \;\left(1-R_{12}^{2}\right)}{A_{2}}\right|\right)\;$$

where d is the diameter of the sample vessel and $R_{12}$ is the reflection coefficient between the buffer rod and the sample.

When Equation (7) is solved for $R_{12}$ and substituted into Equation (10), it results in:

(11) $$\alpha =\frac{1}{2d}\cdot ln\left(\left|\frac{-A_{1}\cdot \;\left(1-{\left(\frac{A_{1}}{C_{0}}\right)}^{2}\right)}{A_{2}}\right|\right)\;$$

### 2.5. Analytical Methods for Sample Assessment

The particle size distribution of all 1 wt% yeast suspensions was determined. In the three corresponding filtrates, extract content, density, alcohol content, osmolality, free amino nitrogen (FAN), and pH were quantified. Ringer solution served as a control reference.

#### 2.5.1. Particle Size Distribution

Particle size distribution of all 1 wt% yeast suspensions was determined by laser diffraction analysis (Mastersizer 3000, Malvern Panalytical Ltd., Malvern, UK). Before measurement, the instrument was calibrated with demineralized water. Subsequently, the 1 wt% yeast suspensions (prepared as described in Section 2.1) were added. Sample measurement time was 20 s, and homogeneous dispersion of the yeast suspension was ensured by using the internal stirrer (stirrer speed = 1200 rpm) and pumping the dispersion through the measuring cell. Particle size distribution measurements were performed in three technical replicates.

#### 2.5.2. Extract, Density, and Alcohol

The extract, density, and alcohol content of the samples were determined with an oscillating U-tube density meter (DMA 4500, Anton Paar GmbH, Graz, Austria). All analyses were carried out in technical triplicates.

#### 2.5.3. Osmolality

Osmolality was measured with a cryoscope for freezing point determination (CryoStar, Funke-Dr. N. Gerber Labortechnik GmbH, Berlin, Germany) according to MEBAK method B-590.06.900 [59]. Three technical replications of each measurement were performed.

#### 2.5.4. Free Amino Nitrogen (FAN)

The FAN content was photometrically determined by measuring the color intensity of Ruhemann’s purple from the ninhydrin reaction according to MEBAK method B-400.11.111 [60]. FAN measurements were performed in three technical replicates.

### 2.6. Statistical Analysis

Statistical analyses were performed using OriginPro^®^ 2023b (OriginLab Corp., Northampton, MA, USA). Normality of the analytical data was examined using the Shapiro–Wilk test (α > 0.05). Significant differences in normally distributed data were identified using one-way analysis of variance (ANOVA, *p* < 0.05). For post-hoc analysis (*p* < 0.05), the Tukey–Kramer HSD-test was conducted for group means [61].

## 3. Results and Discussion

### 3.1. Yeast Suspension Concentration Gradient (0.0–1.0 wt%)—Attenuation Coefficients and Speed of Sound

**Figure 3:**
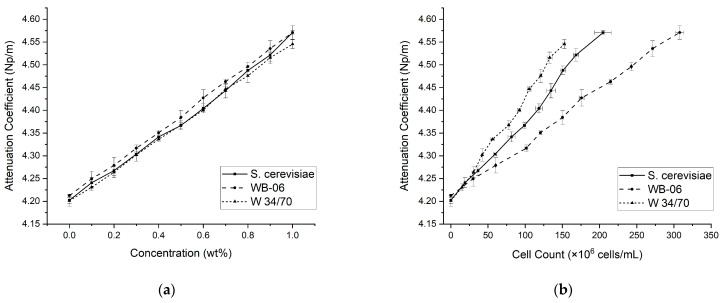
(**a**) Attenuation coefficients (Np/m) of yeast suspensions with concentrations ranging from 0.0–1.0 wt%. (**b**) Correlation between the attenuation coefficient and the yeast cell count. Solid line represents S. cerevisiae, dashed line represents WB-06, and dotted line represents W 34/70. Error bars used for standard deviation (N = 3).

The linear regressions (R^2^ > 0.95), shown in Figure 3a, reveal that the attenuation coefficient increased linearly along the concentration gradient (i.e., 0.0–1.0 wt%) for all three yeast strains. Conversely, although the attenuation of all yeast suspensions progressively rose with increasing cell count, distinct behaviors were observed (see Figure 3b). Compared to S. cerevisiae and W 34/70, the starting cell count of WB-06 is higher and reached maximum concentrations of 307 × 10⁶ cells/mL in 1 wt% suspensions. In contrast, lower cell counts were recorded for S. cerevisiae and W 34/70, reaching maximums of 204 × 10⁶ cells/mL and 152 × 10⁶ cells/mL, respectively. In the 1 wt% suspensions, comparable attenuation coefficients were recorded for all three yeast strains; however, the cell count associated with WB-06 was more than double that of W 34/70, thus indicating the influence of yeast strain properties on the scattering (e.g., yeast cell diameter and volume fraction) and absorption effects (e.g., yeast cell composition and surface properties) of the ultrasound signal. In Figure 3, it can also be observed that within the tested concentration gradient, high precision measurements (σ_max_ = 0.40%) were achieved at all sampling points, further indicating the experimental setup and subsequent data analysis yield consistent, reproducible, and reliable measurements.

**Figure 4:**
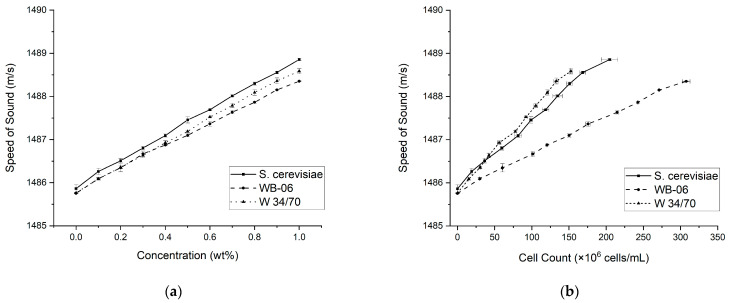
(**a**) Speed of sound (m/s) of yeast suspensions with concentrations ranging from 0.0–1.0 wt%. (**b**) Correlation between the speed of sound and the yeast cell count. Solid line represents S. cerevisiae, dashed line represents WB-06, and dotted line represents W 34/70. Error bars used for standard deviation (N = 3).

The composition of the liquid phase directly influences the speed of sound and was used to characterize the concentration of the dissolved substances in the liquid phase [40,41,42,43,44,45,46,48]. Similar to the attenuation coefficient trends shown above, a linear relationship between the speed of sound and yeast concentration (0.0–1.0 wt%) was also recorded for all three yeasts (R^2^ > 0.95; see Figure 4a). Unlike the attenuation curves in which the values of all three yeasts were within a comparable range, the speed of sound values recorded for the S. cerevisiae concentration gradient is higher and distinctly separated from WB-06 and W 34/70. However, at the highest concentration (1 wt%), the recorded speed of sound was 1488.86 ± 0.09 m/s for S. cerevisiae and closely followed by W 34/70 and WB-06. The speed of sound also increases with the yeast cell count for all three strains (R^2^ > 0.95; see Figure 4b). Similar behaviors are observed in the yeast cell count attenuation trends shown above (Figure 3b) and the speed of sound curves (Figure 4b). The lowest range was measured in the WB-06 samples, whereas the highest speed of sound values was recorded in the S. cerevisiae samples. For all yeast samples, an increased speed of sound was observed at higher cell counts, likely due to changes in the medium’s density and elasticity [62]. The attenuation and the speed of sound of the yeast suspensions were determined with high precision by the used approach. However, further signal processing techniques should be considered. For instance, employing cross-correlation methods could enhance the accuracy of speed of sound measurements [63], and applying zero-padding could increase the frequency resolution of the analysis [64]. Furthermore, hydrophone measurements could improve the estimation of the incident wave amplitude [55].

### 3.2. Comparison of Ringer Solution, Yeast Suspensions (1 wt%), and Corresponding Filtrates

**Figure 5:**
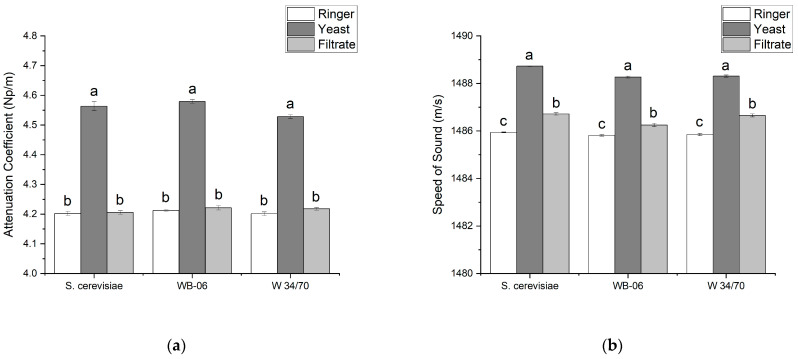
(**a**) Attenuation coefficient (Np/m) and (**b**) Speed of sound (m/s) of Ringer solution (white bars), 1.0 wt% yeast suspension (dark gray bars), and corresponding filtrate (light gray bars). Error bars used for standard deviation (N = 3). Different letters above a bar indicate significant differences in datasets (ANOVA followed by Tukey–Kramer HSD-test, *p* < 0.05). Datasets are grouped by yeast.

To examine the effect of yeast cells on the ultrasound signals, the attenuation coefficients and the speed of sound were determined for each yeast in three separate samples. For each yeast, the evaluated samples included Ringer solution (i.e., 0.0 wt%) as well as unfiltered and filtered yeast suspension at 1 wt%, with the latter referred to as “filtrate”. Figure 5a divides the three samples by yeast (e.g., S. cerevisiae) and compares the measured attenuation coefficients of each group. The composition of all three samples was significantly different. As expected, once the insoluble substances (i.e., yeast cells) were removed, no significant differences (ANOVA, *p* < 0.05) were recorded for the attenuation coefficient of the Ringer solution and the filtrates. Conversely, when compared to the Ringer solution and filtrates, significantly higher attenuation coefficients were measured for all 1 wt% yeast suspensions. The collected data indicate that yeast cells significantly contribute to the attenuation of the ultrasound signal, most likely due to absorption and scattering properties of yeast.

In all three samples, the speed of sound was also measured and once again grouped and compared by yeast (see Figure 5b). The lowest speed of sound values was recorded in all Ringer solution samples and clearly separated from the 1 wt% yeast suspensions. However, unlike the attenuation coefficients, significant discrimination from the speed of sound values of the filtrates was revealed. For all yeasts, the highest speed of sound levels was recorded in the 1 wt% yeast suspensions; these high values are primarily attributed to the presence of yeast cells. Although the speed of sound values recorded for all filtrates are lower than their corresponding 1 wt% yeast suspension, the values are significantly higher than the Ringer solution (i.e., 0.0 wt%); the lowest value was recorded in the WB-06 filtrate. Since visual microscopic assessments of the filtrates were performed, it is confirmed that filtration effectively removed all yeast cells. Therefore, the higher values indicate that other soluble substances influencing the speed of sound remained in the filtrate (see Section 3.4).

### 3.3. Yeast Cell Size Distribution—Dispersed Phase

**Figure 6:**
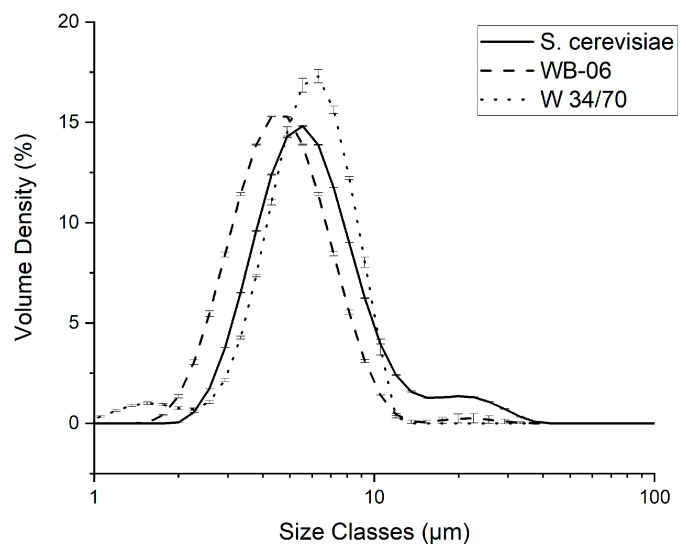
Volume density (%) over particle size (µm) of three different yeasts (1 wt% suspensions). Solid line represents S. cerevisiae, dashed line represents WB-06, and dotted line represents W 34/70. Error bars used for standard deviation (N = 3).

To further characterize the 1 wt% yeast suspensions, particle size (i.e., cell diameter) distribution analyses were conducted. For all yeast strains, the highest volume density peak is detected in the range of 4 to 6 µm (see Figure 6). A characteristic of S. cerevisiae is the bimodal distribution. In addition to the primary peak at 4 to 6 µm, a secondary, smaller peak centered around 20 µm was detected, which may be attributed to clusters of yeast cells. Similar to S. cerevisiae, WB-06 showed a secondary peak centered around 20 µm, indicating that big clusters are a common feature of top-fermenting yeasts. However, a noticeable shift toward the lower-size range is observed for WB-06. The highest peak volume density was recorded for W 34/70. Additionally, the x_50_ value (i.e., median) was calculated from the collected data. The x_50_ value represents the particle size at which 50% of the total particle population is smaller and 50% is larger. The highest x_50_ value was recorded for W 34/70 at 5.80 ± 0.02 μm, followed by S. cerevisiae (5.73 ± 0.06 μm) and WB-06 at 4.61 ± 0.02 μm. As previously mentioned, compared to S. cerevisiae and WB-06, the lowest cell count was detected in the W 34/70 yeast suspension (1 wt%; Figure 3b); however, the highest volume density and cell diameter (x_50_ = 5.80 ± 0.02 μm) were recorded in this sample. Consequently, it can be concluded that the unique properties of the yeast strain, such as particle size distribution, influence the attenuation of the ultrasound signal.

### 3.4. Assessment of Filtrates—Liquid Phase and Dissolved Substances

Detailed chemical and physical properties of the Ringer solution and all 1 wt% yeast suspension filtrates are listed in Table 2. These include extract content, density, alcohol content, FAN, and pH. As previously mentioned, the absence of yeast cells in the filtrates was confirmed by visual microscopic assessment. Of the measured samples, the lowest values for all analyses were recorded in the Ringer solution, except for pH. The highest extract and density were measured in the W 34/70 filtrate, 0.66 ± 0.02%mas, and 1000.70 ± 0.09 kg/m^3^, respectively. Conversely, the top-fermenting yeast, WB-06, yielded the lowest extract and density values. The higher extract and density values in all filtrates are probably attributed to substances that were present during the production of the dry yeast and introduced into the medium through the addition of the dry yeast. Alternatively, these substances were released by the yeast into the medium during rehydration. The alcohol content in all samples is negligible, thus indicating the lack of nutrients in the Ringer solution prevented fermentation from occurring. Further confirming, Ringer solution is an appropriate test medium for ultrasonic measurements of yeast suspensions. Osmolality is a colligative property of a solution, determined exclusively by the concentration of suspended particles, regardless of their size or type [65]. All filtrates showed a significant increase in the concentration of the suspended particles. The osmolality values of both top-fermenting yeasts are in a comparable range, while the highest osmolality was recorded in the W 34/70 filtrate (121.18 ± 0.16 mOsm/kg). Similarly, compared to the Ringer solution, the FAN levels of all filtrates are significantly higher. Increased acidity is confirmed by the lower pH of all yeast filtrates; the lowest pH was recorded at 5.22 ± 0.04 for W 34/70. Compared to the Ringer solution, significant differences in extract content, density, alcohol content, osmolality, FAN, and pH were detected for all yeast filtrates. This is consistent with previous studies, which demonstrated that various substances can co-dissolve with dry yeast, with proteins, amino acids, sugars, and minerals identified as the predominant substances [66,67]. The results in Table 2 suggest that changes in the speed of sound (Figure 4) can be partially attributed to the variation of the liquid phase composition and are directly associated with the concentration of the soluble substances present.

## 4. Conclusions and Outlook

In this study, ultrasound was used to evaluate and compare yeast suspensions prepared from three different *Saccharomyces* yeasts commonly used in industrial fermentation processes. First, the attenuation coefficient and speed of sound in yeast suspensions with increasing concentrations (0.0–1.0 wt%) were measured. For all yeasts, the collected data revealed clear linear relationships between the attenuation coefficient and yeast concentration (R^2^ > 0.95). The yeast cell count was also linearly associated with the attenuation coefficient (R^2^ > 0.95). Similarly, the speed of sound increased linearly with increasing yeast concentration as well as higher cell count. In addition, particle size distribution analyses indicated that volume density and cell diameter influence the attenuation of the ultrasound signal. Conversely, variations in the composition and concentration of soluble substances in the continuous liquid phase of the yeast suspensions had a direct impact on speed of sound values. Compared to the Ringer solution, the filtrates of the 1.0 wt% yeast suspensions showed minor variations in extract content, density, osmolality, FAN, and pH. However, these compositional variations had a statistically significant impact on the speed of sound, resulting in a clear distinction among the Ringer solution and the filtrates. The alcohol content in the filtrates was negligible, yet significantly higher values were recorded for extract content, density, and FAN, as well as the speed of sound. These analyses indicate that soluble substances, which interfere with speed of sound measurements, were either introduced with the yeast or released by the yeast during rehydration. The collected data revealed higher concentrations of the dispersed and liquid phase are associated with higher attenuation and speed of sound. However, the composition of the suspension will directly influence the ultrasound signals. Higher volume density and x_50_ cell diameter of the yeast suspension yield higher attenuation, whereas more soluble substances were associated with higher speed of sound. The ultrasonic data are reproducible and highly precise, further confirming the potential of ultrasound for in-line bioprocess monitoring. The attenuation coefficients and the speed of sound, calculated from the ultrasound signals, can be used to characterize the dispersed solid phase and provide information about the composition of the liquid phase, respectively.

In this study, yeasts were suspended in Ringer solution; the lack of fermentable substances effectively prevented cell metabolism. Future research should also explore the interactive effect of metabolic processes and fermentable sugars on ultrasound signals. In addition, a larger selection of yeast species (e.g., non-*Saccharomyces* yeasts), as well as other fermenting microorganisms, should be examined. Additional aspects to consider are the influence of other relevant fermentation parameters, such as temperature variations as well as nutrient concentrations, on the ultrasound signals. These data could further enhance the robustness and adaptability of the method. The integration of the proposed ultrasound setup should also be tested in industrial bioreactors. Furthermore, ultrasound measurements could be enhanced by integrating advanced machine learning algorithms [68,69]. This integration could result in a non-invasive, in-line, robust, and cost-effective real-time monitoring system for fermentation processes.

## Figures and Tables

**Table 1:** Dry Yeast Strains and Manufacturer Information. The non-italicized code, S. cerevisiae, refers to the *Saccharomyces cerevisiae* samples that were examined.

Code	Strain	Product and Manufacturer
S. cerevisiae	*Saccharomyces cerevisiae*	FERMIPAN^®^ RED, Casteggio Lieviti srl, Casteggio, Italy
WB-06	*Saccharomyces cerevisiae var. diastaticus*	SafAle™ WB-06, Fermentis, Division of S.I. Lesaffre, Marcq en Baroeul, France
W 34/70	*Saccharomyces pastorianus*	SafLager™ W 34/70, Fermentis, Division of S.I. Lesaffre, Marcq en Baroeul, France

**Table 2:** Mean and Standard Deviations (N = 3) of Extract Content, Density, Alcohol, Free Amino Nitrogen (FAN), and pH of Ringer Solution and Filtrates of 1 wt% Yeast Suspensions. Different superscript letters in a column indicate significant differences in datasets (ANOVA followed by Tukey–Kramer HSD-test, *p* < 0.05).

		Extract [%mas]	Density [kg/m^3^]	Alcohol [%mas]	Osmolality [mOsm/kg]	FAN [mg/100 g]	pH
	Ringer	0.52 ± 0.00 ^c^	1000.23 ± 0.00 ^c^	0.00 ± 0.00 ^c^	105.33 ± 0.61 ^c^	0.00 ± 0.00 ^c^	6.90 ± 0.03 ^a^
**Filtrates**	S. cerevisiae	0.64 ± 0.01 ^a^	1000.63 ± 0.03 ^a^	0.03 ± 0.00 ^a^	116.87 ± 0.15 ^b^	8.69 ± 0.18 ^a^	5.41 ± 0.02 ^c^
	WB-06	0.60 ± 0.01 ^b^	1000.50 ± 0.04 ^b^	0.02 ± 0.01 ^b^	116.47 ± 0.51 ^b^	7.79 ± 0.35 ^b^	5.51 ± 0.02 ^b^
	W 34/70	0.66 ± 0.02 ^a^	1000.70 ± 0.09 ^a^	0.02 ± 0.00 ^b^	121.18 ± 0.16 ^a^	8.95 ± 0.51 ^a^	5.22 ± 0.04 ^d^

## Data Availability

The data that support the findings of this study are available from the corresponding author upon reasonable request.
